# Quantifying the recarbonization of post-agricultural landscapes

**DOI:** 10.1038/s41467-023-37907-w

**Published:** 2023-04-14

**Authors:** Stephen M. Bell, Samuel J. Raymond, He Yin, Wenzhe Jiao, Daniel S. Goll, Philippe Ciais, Elsa Olivetti, Victor O. Leshyk, César Terrer

**Affiliations:** 1 Department of Civil and Environmental Engineering, Massachusetts Institute of Technology, Cambridge, MA 02139 USA; 2 Laboratoire des Sciences du Climat et de l’Environnement, LSCE/IPSL, CEA-CNRS-UVSQ, Université Paris-Saclay, 91191 Gif-sur-Yvette, France; 3 Institute of Environmental Science and Technology, Universitat Autònoma de Barcelona, 08193 Bellaterra, Spain; 4 MIT Climate and Sustainability Consortium, Cambridge, MA 02139 USA; 5 Department of Geography, Kent State University, 325 S. Lincoln Street, Kent, OH 44242 USA; 6 Department of Materials Science and Engineering, Massachusetts Institute of Technology, Cambridge, MA 02139 USA; 7 Center for Ecosystem Science and Society, Northern Arizona University, Flagstaff, AZ 86011 USA

**Keywords:** Carbon cycle, Restoration ecology, Climate-change mitigation, Carbon cycle

## Abstract

Despite worldwide prevalence, post-agricultural landscapes remain one of the least constrained human-induced land carbon sinks. To appraise their role in rebuilding the planet’s natural carbon stocks through ecosystem restoration, we need to better understand their spatial and temporal legacies.

In provisioning human civilization with food, fuel, and fiber for millennia, agriculture has drastically depleted terrestrial carbon stocks at the expense of natural ecosystems. Our challenge today is to use more sustainable practices to recapture some of the 116 Pg of soil organic carbon (SOC) lost since agriculture began, while simultaneously ensuring global food security^1,2^. That being said, the cessation of agriculture altogether is still the most efficient way to increase carbon stocks and restore ecosystems in tandem and at large scales.

Consider the vast expanses of forests that regrew over the 60 Mha of cropland abandoned following the collapse of the Soviet Union^3^. It has been called the world’s largest human-made carbon sink attributed to a single event;^4^ a title challenged by the climatic consequences of the ‘Great Dying in the Americas’ and its 56 Mha abandoned following the arrival of Europeans^5^. At more practical scales, intentional efforts to restore agricultural land such as the Grain-for-Green program in China and the Conservation Reserve Program in the USA have demonstrated that carbon sequestration is far from being the only advantage^6,7^. Ecological co-benefits include reduced soil erosion and water run-off, reduced flooding and drought, and improved soil health, water quality, and biodiversity indicators.

These post-agricultural landscapes (PALs) often signify the return of ecosystem properties, such as carbon, towards pre-disturbance states or new equilibria through secondary succession. Whether planned or unplanned, they appear in every agricultural region of the world and they can drawdown carbon with or without human involvement. If commitments to halt gross forest area loss by 2030 succeed, recarbonizing PALs will play a key role in reversing global land use change from being a net carbon source to a net sink^8^.

Unfortunately, PALs are insufficiently represented in terrestrial carbon models, both spatially (as a poorly mapped land cover class) and temporally (as uncertain carbon sinks). This hinders our ability to monitor, quantify, and leverage them strategically. We discuss here some of the reasons behind these issues and what can be done to address them so that we can properly evaluate the role of PALs.

## Challenges in conceiving PALs as carbon sinks

The first challenge with PALs is that they are diverse and heterogeneous; categorizing them as a single land cover class is not easy. They include several overlapping terms, some used interchangeably (e.g., old fields, idle croplands, set-aside), and others used only in specific cultural contexts (e.g., post-agrogenic lands in the former-Soviet states). Some PALs also have negative connotations, creating difficulties for policy-makers, local stakeholders, and landowners to agree on how to manage them.

Abandoned agricultural lands are probably the most well-known PAL, but “abandonment” can mean different things. In many cases, it is perceived positively as an opportunity for ecosystem regeneration and renaturalization, while for others it signals local economic depression, the loss of traditional rural livelihoods and biodiverse agro-landscapes, and an increased risk of wildfires and pests when the land is left unmanaged^9^. Complicating things further, there is still no universal definition of abandonment with an agreed minimum time frame. The Food and Agriculture Organization considers agricultural land to be abandoned after five consecutive years of disuse, but this rule is not universally applied.

Another challenge is that any sequestration and ecological co-benefits PALs may produce can be rapidly reversed through recultivation. Of course, many PALs are not permanent by definition and were never meant to be, such as lands under fallow or shifting agriculture. But others are simply unprotected: abandoned agricultural lands are at constant risk of recultivation in their first few decades and often before significant ecological benefits are realized^10^.

These issues notwithstanding, arguably the biggest roadblock in protecting, monitoring, and utilizing PALs as carbon sinks is finding them. While agricultural lands intentionally converted to pre-disturbance states (i.e., cropland restoration) likely entail an administrative record that can be used to validate and update spatial estimates, other kinds of PALs emerge unannounced all over the world every day. Agricultural land abandonment often goes undeclared unless incentives are present and governmental bodies are involved.

Insufficient data on the different drivers of agricultural cessation—which are numerous^11^—further hinders efforts to map past, present, and predicted PALs. Even if we do map them well, we still need to figure out long they have existed to be able to evaluate their ecological impacts accurately. Remote sensing produces our best estimates of PAL distributions at large geographic scales, but only for the past few decades and with high uncertainties depending on the type of PAL investigated and the methods used.

Finally, the rates of carbon sequestration on PALs are also largely unresolved. We know multiple factors modulate SOC and biomass accumulation following agricultural cessation, such as the past and present management practices and their legacies (e.g., historical fertilization rates, crop types cultivated, etc.), soil and vegetation properties, climate, and, of course, time. However, we have yet to unravel how different combinations of these factors might explain the diversity of positive, negative, or negligible SOC changes reported on PALs globally.

Better data, and better use of existing data, is desperately needed to clarify underlying relationships, model PAL impacts in all agricultural regions of the world, and ultimately predict potential carbon sinks and sources through time. Unfortunately, the gold standard of repeated measurements and long-term experimental field sites not only takes too long, but is logistically and financially prohibitive for many researchers. We remain severely limited in our ability to promote PALs as components of sustainable land management strategies.

## Constraining the carbon sink potential of PALs

Since there is still no consensus on the global extent of PALs, the first step to constrain their carbon sink potential is to produce reliable, up-to-date, spatial estimates of past, present, and predicted instances of agricultural cessation. Even successful mapping approaches struggle in some situations because PALs can involve the entire spectrum of agricultural land types, plot sizes, and management practices^12^. For example, it is extremely difficult to distinguish between abandoned pastures and natural grasslands with current remote sensing methods. Agricultural cessation in small fields (i.e., < 0.1 ha) are often beyond the detection limits of open-access imagery such as NASA/USGS’s Landsat or MODIS archives.

Fortunately, our ability to detect and classify PALs is improving. Higher spatial, temporal, and spectral resolution remote sensing is now available with the denser time series provided by the ESA’s Sentinel-1 and Sentinel-2, together with Landsat data. There are also increasing options for very high-resolution (e.g., < 5 m) commercial satellite imagery that can be used to isolate and process smaller agricultural plots and distinguish between different agricultural management practices (e.g., PlanetScope)^13^. Aside from satellites, platforms like IIASA’s Geo-Wiki (www.geo-wiki.org) demonstrate the potential of crowd-sourced ground truthing to supplement visual interpretations in training land cover classification models.

The large volume of data generated by new sensors can also be processed more rapidly and with more accessibility than ever before using free cloud computing resources like Google Earth Engine^14^. Combining these approaches with advances in machine learning algorithms being continuously developed with free, open-source software libraries has already produced new land use and land cover products with unprecedented spatiotemporal resolutions (e.g., Dynamic World)^15^. Although significant challenges still exist in upscaling ecological properties like SOC globally^16^, we should anticipate the first reliable maps of historical and existing PALs (and their durations of existence) and prepare for their integration into terrestrial carbon cycle research.

Therefore, simultaneous with mapping efforts, we also need to address the lack of temporal SOC data on PALs. Time-stamped data points are crucial for understanding what factors determine if and when a given PAL will act as a carbon sink or source following agricultural cessation. Currently, global biogeochemical models are limited by relatively poor temporal data (i.e., low quality and quantity) due to the logistical and financial challenges of long-term field sites with repeated measurements, especially in under-resourced regions^17^.

**Fig. 1:**
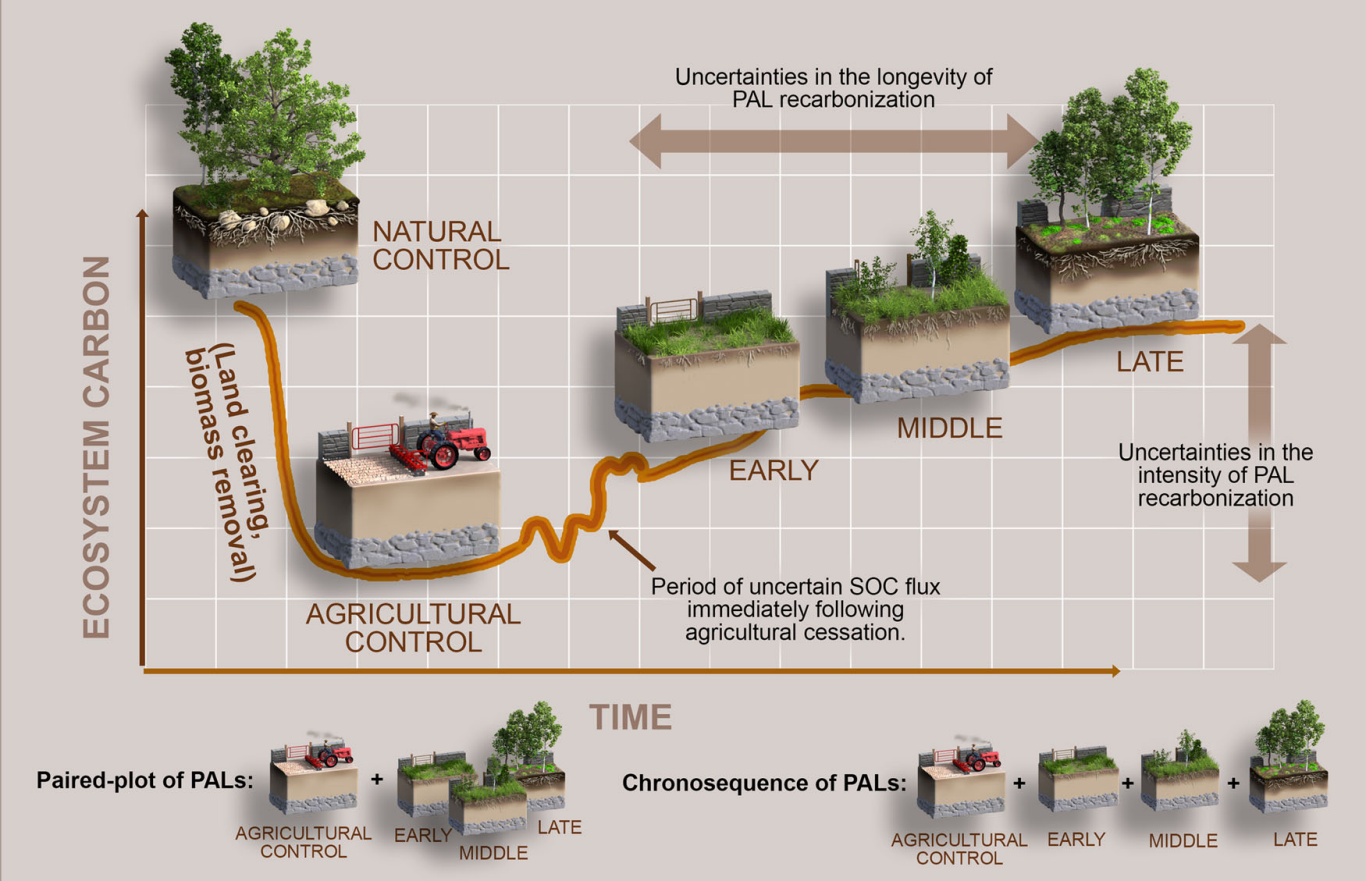
Typical post-agricultural landscape (PAL) trajectory involving land clearing from the primary forest (natural control) for agricultural use (agricultural control) and ecological succession following agricultural cessation (shown here as early-, middle-, and late-stage PALs). The rebuilding of ecosystem carbon stocks in PALs is represented by the increasingly darkening top soils and growing plant biomass. However, the ecological and structural legacies of land clearing, tilling, and other destructive practices have lasting impacts on above- and below-ground properties, preventing a rapid return to pre-disturbance states. Instead of sampling the same PAL over time (i.e., repeated measurements), a quicker and less expensive alternative is to compare samples from an active agricultural plot and one (i.e., with the paired-plot method) or more (i.e., with the chronosequence method) PALs that share the same environmental and management characteristics except for the time since agricultural cessation. When sampled simultaneously and analyzed sequentially, these plots can reveal the temporal dynamics of ecosystem carbon. Active agricultural plots represent the expected baseline of all the plots before agricultural cessation, while natural control plots can be used to represent pre-disturbance states and, therefore, idealized PAL end-states. Not only are the logistical barriers to paired-plots and chronosequences much lower than repeated measurements, and so they should be established widely to fill data gaps, but existing published data using these approaches are also a largely untapped resource for benchmarking successional carbon modeling.

Thankfully, space-for-time substitutions like paired-plots and chronosequences are accessible, inexpensive, and rapid alternatives that can help fill the many geographical gaps in temporal ecosystem data on PALs (Fig. 1). Although they have notable limitations and potential sources of error (e.g., inappropriate control site selection), they are logistically sound and generally informative for exploring broad ecological processes in lieu of long-term repeated measurements^18^. Chronosequence and paired-plot sampling in underrepresented PAL regions should be especially prioritized to fill critical data gaps (i.e., South America, Africa, and most of Asia based on our global survey of > 3400 published SOC data pairs from PALs).

Investing new time, money, and energy may not even be necessary for agricultural areas that are already densely sampled. Previously published paired-plots and chronosequences studies of PALs featuring carbon data are numerous, readily available, and valuable sources of data that can complement well-known, widely used datasets of long-term repeated measurements. They represent past investments in science that should be repurposed, reused, and archived before being lost^19^. Valuable secondary soil physicochemical properties (e.g., nitrogen, phosphorus, particulate and mineral-associated organic matter, etc.) for modeling drivers of SOC dynamics are also often available within the same study. Collecting this information can improve our inferential capabilities and reduce geographic and statistical biases.

By extracting and synthesizing disparate time-stamped SOC and auxiliary data from published studies involving paired-plots and chronosequences of PALs, in addition to sampling new sites in underrepresented regions, we can benchmark and validate models of successional carbon dynamics. Widely referenced global syntheses that include categories analogous to recarbonizing PALs are outdated and severely data-poor^20,21^. More recent regional studies still lack sufficient temporal resolution, site-specific parameters, and management information to allow for upscaling. A robust, updated global synthesis exploring recarbonizing PALs is overdue.

## Leveraging PALs as carbon sinks

Integrating better maps and bigger time-stamped datasets with new approaches in artificial intelligence-driven modeling will allow us to investigate the carbon cycle impacts of PALs with more accuracy than ever before. Degraded and marginal agricultural lands already represent some of the best candidates for ecosystem restoration globally. These research efforts will provide critical and timely insights for international reforestation and carbon sequestration initiatives.

Whenever and wherever PALs do recarbonize, regardless of their carbon sink longevity and permanence, they must be made known and accounted for ref. ^22^. Of course, not all PALs can be expected to recarbonize naturally. Those that are degraded beyond spontaneous recovery will need a helping hand through assisted restoration practices like native tree planting. Afterall, mitigating the negative impacts of climate change by returning carbon to depleted soils will require the use of multiple avenues simultaneously^23^.

**Fig. 2:**
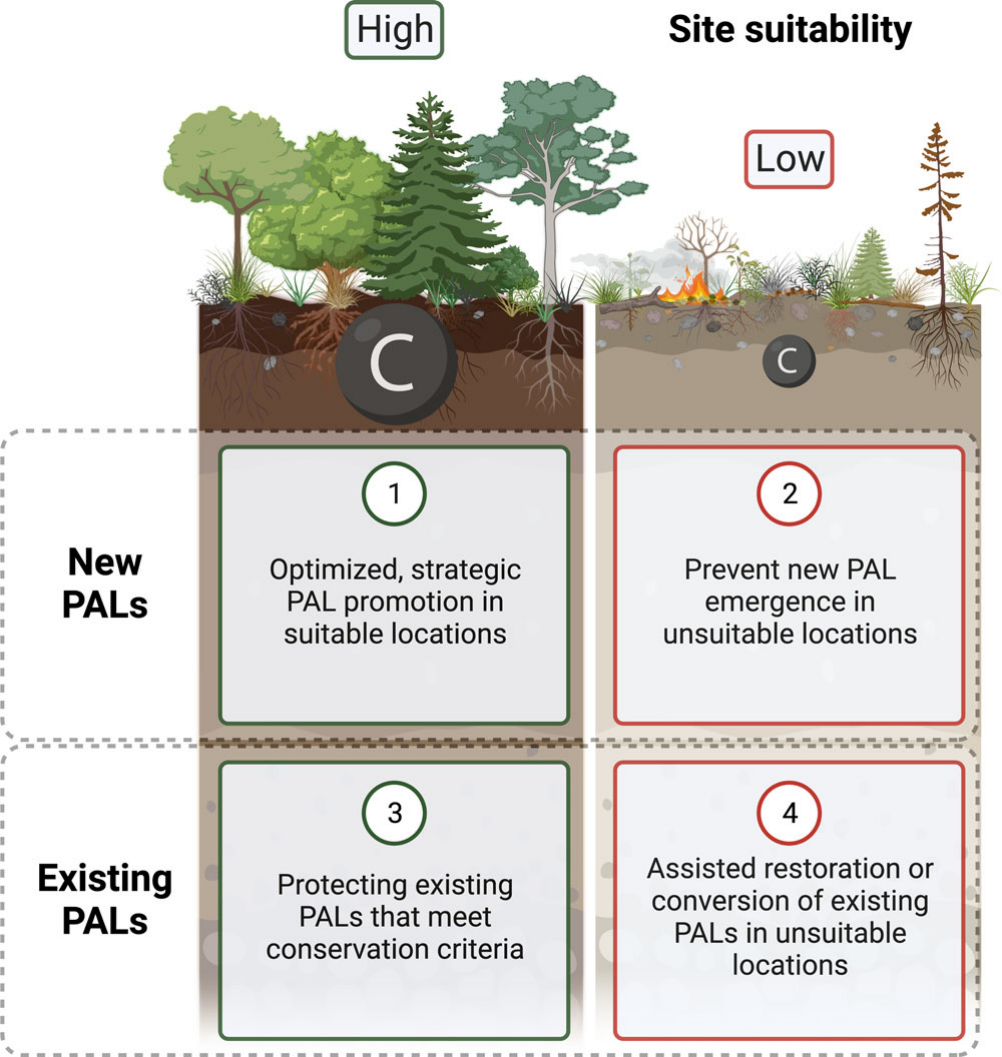
The suitability of an agricultural site will determine if and how it should be managed as a recarbonizing post-agricultural landscape (PAL). New and existing PALs can have a high or low potential for soil and ecosystem carbon (C) sequestration and high or low feasibility when considering various competing needs (e.g., food production), trade-offs (e.g., wildfire risk), and conflicts (e.g., local land rights). Depending on the carbon data available, the definitions used, and the land use factors considered, possible land management trajectories include (1) implementing or (2) preventing new PALs and (3) protecting or (4) restoring or converting existing PALs into more appropriate land uses. Increased temporal data and improved spatial estimates of PALs will support decision-makers in navigating scenarios like these. This graphic was created with BioRender.com.

Ultimately, the identification of suitable locations for recarbonizing PALs will enable decision-makers to weigh trade-offs and consider different land management trajectories (Fig. 2). Optimized promotion of PALs as carbon sinks might entail incentivizing landowners and managers in suitable locations to maintain existing PALs or create new PALs through agricultural cessation. Payment for predicted and validated carbon sequestration, as one example, would help deter farmers from seeking alternative economic pathways for their land that would result in lost opportunities for climate change mitigation.

Future discussions at the international level on promoting PALs as carbon sinks must carefully account for potential conflicts with food production, biodiversity, rural socioeconomic needs, and other sustainable development goals. Land tenure can be particularly unclear in regions with PALs^24^; ensuring fair and equal land rights considering broad categories of stakeholders is a significant challenge. But most importantly, any agricultural land expansion as a result of PAL promotion should be monitored and strictly prevented. It would risk negating any benefits realized and become another example of carbon leakage if payments for ecosystem services are involved.

Uncontested PALs would be ideal candidates for conservation^25^. For contested PALs, it is the responsibility of the research community to provide accurate and reliable carbon sink data for decision-makers to weigh amongst all other competing priorities. Without this information, PALs will continue to be just another uncertain and ultimately misused piece of the sustainable land management puzzle.

It is unreasonable that in many agricultural regions we still cannot directly compare the potential costs, benefits, and synergies of sustainable practices (e.g., regenerative agriculture) with the cessation of agriculture altogether. Quantifying recarbonizing PALs will help advance regional debates on land sharing versus land sparing by filling critical data gaps on the restoration, and in some cases rewilding, of agricultural lands.

PALs can diversify the global land sink and provide timely support for the UN Decade of Ecosystem Restoration (2021–2030) by fulfilling local- to global-level commitments to reverse ecosystem degradation. But they are in need of an immediate, concerted effort by researchers to appraise their role in the terrestrial carbon cycle. PALs are widespread, carbon-depleted, and often neglected, despite periodic calls for conservation and reutilization^26,27^. Let’s make this the last call.

It’s time to map, model, and manage the world’s best PALs for carbon sequestration and ecosystem restoration.
